# Incidence of *Aeromonas* spp. infection in fish and chicken meat and its related public health hazards: A review

**DOI:** 10.14202/vetworld.2016.6-11

**Published:** 2016-01-02

**Authors:** Praveen Kumar Praveen, Chanchal Debnath, Shashank Shekhar, Nirupama Dalai, Subha Ganguly

**Affiliations:** 1 Department of Veterinary Public Health and Epidemiology, Faculty of Veterinary and Animal Sciences, Kolkata - 700037, West Bengal, India; 2 Department of Animal Genetics and Breeding, Faculty of Veterinary and Animal Sciences, Kolkata - 700037, West Bengal, India; 3 Department of Veterinary Physiology and Biochemistry, Faculty of Veterinary and Animal Sciences, Kolkata - 700037, West Bengal, India; 4 Department of Fish Processing Technology, Faculty of Fishery Sciences, West Bengal University of Animal and Fishery Sciences, Kolkata - 700037, West Bengal, India

**Keywords:** *Aeromonas*, antibiotic resistance, chicken, fish, public health

## Abstract

*Aeromonas* is recognized to cause a variety of diseases in man. In humans, they are associated with intestinal and extra-intestinal infections. With the growing importance of *Aeromonas* as an emerging pathogen, it is important to combat this organism. It is indisputable that *Aeromonas* strains may produce many different putative virulence factors such as enterotoxins, hemolysins or cytotoxins, and antibiotic resistance against different antibiotics. The ability of these bacteria to grow competitively at 5°C may be indicative of their potential as a public health hazard. Comprehensive enteric disease surveillance strategies, prevention and education are essential for meeting the challenges in the years ahead. It is important for us to promote the value of enteric cultures when patients have a gastrointestinal illness or bloody diarrhea or when multiple cases of enteric disease occur after a common exposure. With the growing importance of *Aeromonas* as an emerging pathogen, it is important to combat this organism. It is indisputable that *Aeromonas* strains may produce many different putative virulence factors, such as enterotoxins, hemolysins or cytotoxins. It has been established that aerolysin is a virulence factor contributing to the pathogenesis of *Aeromonas hydrophila* infection. Fish and chicken play an important role in the transmission of this pathogen to humans. In the present study, the high prevalence of toxin-producing strains was found among the *Aeromonas* isolates. The ability of these bacteria to grow competitively at 5°C may be indicative of their potential as a public health hazard. The present review was constructed with a view to highlight the zoonotic importance of *Aeromonas* pathogen in fish and chicken meat.

## Introduction

In the last two to three decades *Aeromonas* spp. have emerged as an important human pathogen. Praveen *et al*. [1] conducted a study on 179 samples from fish (Gills), and chicken (Raw meat) were processed for bacteriological examination. Ampicillin dextrin agar (ADA) media was used for isolation of aeromonads from fish and chicken. A total of 31 (17.32%) isolates were obtained from 179 samples of fish and chicken in the North Kolkata Region. In 31 isolates, the highest isolation were achieved from fish 24 (18.89%), followed by chicken 7 (13.46%) out of which 29 (93.54%), 1 (3.22%), 1 (3.22%) were recognized as three species of *Aeromonas* namely *Aeromonas hydrophila*, *Aeromonassobria*, and *Aeromonascaviae*, respectively, through biochemical characterization including aesculin hydrolysis, Voges–Proskauer (VP) and gas from glucose (triple sugar iron) tests. The organism of this genus is oxidase and catalase positive, facultatively anaerobic, Gram-negative, short rod-shaped bacteria. The highly valuable fish and chicken are not always safe to consume from the public health point of view. The newly identified etiological agents of diarrheal disease are mesophilic aeromonadswhich have emerged as an important public health hazard [2]. There is mounting evidence of their involvement in gastrointestinal and extraintestinal infection in human beings [3,4]. The genus *Aeromonas* comprises of two different groups of bacteria. One is non-motile psychrophilic *Aeromonas salmonicida* and the other group comprising of three mesophilic motile spp. *A. hydrophila*, *A. caviae*, and *A. sobria* [5]. *Aeromonas* is an environmental microorganism. They are cosmopolitan in distribution. Mesophilic motile aeromonads are ubiquitous and autochthonous aquatic microorganisms occurring in fresh water, sewage and brackish water [6] and in chlorinated and unchlorinated drinking water [7,8]. Besides these aeromonads occur as the normal microbial flora of many aquatic and terrestrial animals and are proven diseases in various cold blooded and warm blooded animals including fishes, amphibians, reptiles, birds and other domestic animals [9,10]. Not only the aquatic environment plays an important role in the dissemination of aeromonads, different foods, especially, fishes and other seafood, raw and cooked meat,chicken, vegetables, milk and milk products can be a potential vehicle for human infections as well as animals [11-16]. The present review discusses and elaborates on the zoonotic importance of *Aeromonas* pathogen in fish and chicken meat.

## Isolation and Identification of Aeromonas

Fricker and Tompset [13] in a study examined 563 samples of fish, raw and cooked meats and of pre-prepared salads from retail outlets and reported the isolation of mesophilic *Aeromonas* spp. from 287 samples and the most frequent contaminated samples were chicken (79.3%) and offal (84.3%). *A. salmonicida* was isolated from paddlefish (*Polyodon spathula*) during an epizootic of furunculosis in river state Hatcheries, Arkansas [16]. The isolates were obtained from cultures of gills and kidney tissues [17]. *Aeromonas* spp. Were isolated from gills, eggs, stomach and ventral muscles of fresh water prawns available in the local fish market of Dhaka, Bangladesh [18]. A study on food samples including fish was carried out and reported that out of 382 food samples 40 (10.47%) were positive for aeromonads. Out of 99 fish samples 22 (22.22%) were found positive, in which *A. hydrophila* (66.6%), *A. sobria* (27.27%), *A. caviae* (9.09%) were found positive [19]. An examination was done on 68 food samples for the presence of mesophilic *Aeromonas* species both qualitatively and quantitatively. Aeromonads were isolated from 26% of the vegetable samples, 70% of the meat and chicken samples and 72% of the fish and shrimps [20]. One reportof isolation of a total of 319 strains of *A. hydrophila* from 536 fish and 278 prawns for a 2-year period survey and the samples were collected from a major fish market of Coimbatore, Tamil Nadu, South India [18]. There was the detection of 82 strains of presumptive *Aeromonas* spp. out of a total of 250 frozen fish (Tilapia) samples purchased in local markets in Mexico City [21]. A study of 87 fish samples was done and reported that out of 87 fish samples 60 (69%) were found positive for *Aeromonas* species. Out of 60 isolates obtained, *A. veronii biovar sobria* 48, *A. hydrophila* 10 and *A. caviae* were found in 2 isolates [22]. In an examination of total of 78 raw and 123 processed and ready to eat retail food samples to assess the presence of motile *Aeromonas* spp. with the conventional cultivation method based on the use of ADA medium. He reported 65/201 (32.3%) samples showed presumptive *Aeromonas* colonies. The rate of contaminated samples and the presence of pathogenic *Aeromonas* reported by him were significantly lower for processed than in the case of raw samples [23]. A study was carried out on isolation and identification of motile *Aeromonas* spp. from 53 samples of raw chicken meat. They found 47.17% of the sample to be positive for motile *Aeromonas* spp. and of this 28.30% represented *A. hydrophila* and 9.43% of *A. sobria*, thus stating that the presence of these pathogenic organisms in the raw chicken samples possessed a serious potential risk for public health [24]. In an examination of 332 food samples of chicken, fish, pork and chevon, out of 332 food samples, 38 (11.44%) food samples were positive for *Aeromonas* species. In the case of fish out of 137 samples, 18 (13.13%) and in chicken out of 104 samples, 12 (11.53%) were found positive for *Aeromonas* species [25]. A study were carried out on 154 food samples (chicken, fish and ready-to-eat sprouts) from different retail outlets in Mumbai, India. They analyzed that out of 154 food samples tested, 22 (14.28%) isolates were *Aeromonas* species. The highest percentage of isolation were from chicken (28.6%), followed by fish (20%) and sprout (2.5%) [26]. In an examination 53 (57.6%) isolates of aeromonads from 92 chicken samples and 27 (17%) isolates of aeromonads from 158 minced meat samples. The isolation rate in chicken was significantly higher than minced meat. The highest contamination was found in chicken with *A. caviae* and minced meat contaminated with *A.hydrophila* [27-31].

## Antibiotic Resistance

McNicol *et al*. [32] carried out a study on antibiotic-resistant strains of *A. hydrophila* from aquatic environments of Bangladesh. These strains carried resistance to chloramphenicol, streptomycin and tetracycline. A study was done on the activity of ß-lactam antibiotics upon 20 strains of *A. hydrophila* [29]. Higher degrees of resistance to ampicillin and cephaloridine were observed along with the highest activity of clavulanic acid when assayed at sub-inhibitory concentrations in association with ampicillin [29]. One examination was performed on minimal inhibitory concentrations of 22 antimicrobial agents for 60 strains of three *Aeromonas* spp. by microdilution method. They found that newer cephalosporins such as moxalactam, cefotaxime, and cefoperazone, the aminoglycosides and chloramphenicol, tetracycline, nitrofurantoin and trimethoprim-sulfamethazole inhibited most of the strains within the genus and *A. hydrophila* was more resistant than either *A. caviae* or *A. sobria* to the antibiotics tested [30]. A studywas carried out on the scope of antibacterial resistance among *Aeromonas* spp. from a variety of tropical fish species imported from Singapore [31]. There were 34 *A. hydrophila* strains from various fish and geographical locations isolated to study their antibiotic resistant pattern. It was observed that all the strains possessed multiple resistance, most commonly ampicillin and carbenicillin, whereas rifampicin was the most active antibiotic amongst these strains [32]. 80 isolates of *A. salmonicida* were isolated from separate outbreaks of furunculosis in farmed and wild salmon in Scotland during 1988-1989 and all these isolates subjected to susceptibility to ß-lactam antibiotic amoxycilline were found to be resistant [33]. In a collection of 234 isolates of *Aeromonas* primarily *A. hydrophila* the increasing antibiotic resistances in Taiwan was studied. By agar dilution method, they found that more than 90% of the isolates were susceptible to moxalactam, ceftazidine, cefepime, aztreonam, imipenem, amikacin and fluoroquinolones, but more resistant to tetracycline, trimethoprim-sulfamethoxazole, cephalosporins and aminoglycosides [34]. In a reportgentamicin, chloramphenicol, ciprofloxacin and cotrimoxazole were sensitive to *Aeromonas* isolates [19]. A study was carried out on surveillance of bacterial susceptibility to five antimicrobial agents during 1-year period demonstrating high levels of individual and multiple antimicrobial resistances among the aeromonads. Here-forth indicating a substantial impact of fish farming on several groups of bacteria associated with aquaculture environment [35]. In a report of antibiogram studies, observation was found that chloramphenicol were highly sensitive to *Aeromonas* species [22]. In a study on antibiotic susceptibility of *A. hydrophila* and *A. sobria* isolated from farmed carp (*Cyprinus carpio*) and observed that of 21 isolates examined 100% were resistant to ampicillin and penicillin and sensitive to trimethoprim-sulphamides, oxolinic acid, flumequine, chloramphenicol, norfloxacin, lincomycin, and perfloxacin [36].

There were 132 *Aeromonas* isolated from the fish market in Ankara, turkey; predominant being *A. cavaie* (66%) followed by *A. hydrophila* (22.6%) and *A. veroniibiovarsobria* (11.6%). All the aeromonads were found to be resistant to ampicillin, cephalothin and trimethoprim, but least resistant to chloramphenicol (9.0%) and in contrast, all the above strains were susceptible to ciprofloxacin and ceftriaxone [37]. An experiment was done on 51 strains of *Aeromonas* isolates from 20 rainbow trouts (*Oncorhynchus mykiss*) to determine the sensitivity to different groups of ß-lactam antibiotics (penicillin, cephalosporins, monolactams, and carbapenems) through disc diffusion method and found that highest rate of resistance was to ampicillin, carbenicillin and ticarcillin [38]. A study was carried out on the occurrences of resistance to antimicrobials from a variety of aquacultural species. It was observed that resistance to ampicillin, amoxicillin, cephalexin and erythromycin was widespread, but all the strains were highly susceptible to ciprofloxacin [39]. A survey on fish rearing in a prototype marine integrated a system where oxolinic acid treatment was given and the level of resistance to quinolone antibiotic monitored. In their result, they observed that a resistance had occurred in the intestines of fishes undergoing such treatment in contrast to no evolution of resistance level either in bivalves or in sediments of the integrated acquatic system [40]. An antibiotic sensitivity test report was shown on *Aeromonas* that *Aeromonas* were sensitive toward ciprofloxacin, streptomycin, amikacin and gentamicin [41]. A study was carried out on antibiotic sensitivitytest on 22 isolates of Aeromoas. The study reported that *Aeromonas* isolates were sensitive to gentamicin, ceftrixone and chloramphenicol [42]. In an examination antibiotic resistance pattern shown that more than 90%, 80%, 70% and 60% of the strains were resistant to ampicillin, cephalethrin, tetracycline and nalidixic acid, respectively [43].

## Virulence

In a reportof virulence study, the report suggested that the spoilage potential and pathogenicity of *Aeromonas* spp. in shellfish and food correlated well with its ability to secrete several extracellular virulent factors such as hemolysin, enterotoxins, cytotoxins, lipase and protease [34]. There were 40 strains of motile *Aeromonas* isolated from healthy fish and identified them as *A. hydrophila* and *A. sobria*. They observed that strains of *A. hydrophila* only produced a dermonecrotic factor and two zones of hemolysis on blood agar and 72% of them were enterotoxigenic [35]. In acomparisonof biochemical characteristics and virulence factors in 147 *Aeromonas* spp. isolated from environmental sources. 91% of the isolates were enterotoxigens and produced hemolysins, thus, suggesting that such isolates may be a source of enteric pathogens [44]. A study showed the significance of *Aeromonas* spp. as potential water-borne enteric pathogens by their biochemical characteristics and virulence-associated properties that are hemolysin and enterotoxin [45]. The majority of the enterotoxigenic strains isolated were *A. sobria* which exhibited properties of VP positive, arabinose negative and lysin decarboxylase positive [45]. An examination characterized 73 *Aeromonas* strains to species level and examined for their ability to produce virulence factors. They observed that strains identified as *A. sobria* were the strongest producers of hemolysin and enterotoxin, whereas *A. caviae* strains were consisitently non-hemolytic and non-enterotoxigenic [46]. A study characterized 58 *Aeromonas* strains from patients with or without diarrhea in the Philippines. Out of 58 *Aeromonas* strains, 26% were enterotoxigenic, 41% produced cytotoxin and 51% produced hemolysin. Of the three spp. (*A. hydrophila, A. caviae* and *A. sobria*) studied, *A. sobria* was the most toxigenic with 22 of 30 exhibiting atleast one toxigenic property [47]. In an examination *A. hydrophila* from food and drinking water isolated and tested their pathogenicity by studying the properties related to hemolysis, haemagglutination and cytotoxicity [48]. They suggested that human intestinal cell line HT-29 would be a useful complement for studying the enteropathogenicity of the species for humans [48]. A study wascarried out on 6 tests (4 *in-vitro* and 2 *in-vivo*) and reported that 77.5% of isolates were positive for β-hemolysis and 7.5% for α-hemolysis. Out of 77.5% *A. hydrophila* (70.53%), *A. sobria* (92.30%) and *A. caviae* (66.66%) showed hemolytic activity [19]. In Norway, an examination was done on 31 isolates of *Aeromonas* spp. Isolated from food and water for the possible virulence factors. They recorded five different species, *A. caviae* (9/31), *A. hydrophila* (15/31), *A. schubertii* (3/31) and *A. veronii* biovar *veronii* (1/31), of which *A. hydrophila* were found to be responsible for small outbreaks of food poisoning caused by ingestion of raw fermented fish. All the strains produced and secreted cytotoxins at 37°C [49-52]. A result indicated that potentially enteropathogenic *Aeromonas* were commonly present in untreated drinking water and had been associated with intestinal and extraintestinal infections in humans [50]. A research was carried out to detect the incidence of motile *Aeromonas* in a variety of raw meat products consumed commonly in Ankara, Turkey. They found that *A. hydrophila* and *A. sobria* were the dominant species and strong producers of hemolysin and all the aeromonads were highly susceptible to ciprofloxacin (100%) but resistant to ampicillin and erythromycin (100%) [51]. In a study report the identification of *A. sobria* (67.3%) and *A. hydrophila* (21.2%) in meat and offals sold in Port Harcourt, Nigeria was reported. They found all the strains to be producers of hemolysin and these isolates were all susceptible to chloramphenicol and resistant to ampicillin. They also reportedthat aeromonads were unlikely to pose public health problems in Nigeria as meat undergoes prolonged cooking procedures [51]. In an examination result, it was concluded that all isolates were found to be associated with at least one virulent gene. Moreover, they were resistant to frequently used antibiotics for human infections. The study revealed that the pathogenic potential of *Aeromonas*, associated with ornamental fish culture systems suggesting the emerging threat to public health [52].

Earlier study [53] indicated hemolysin production in a lesser percentage of the isolates compared to the results obtained in this study [5]. A majority of hemolytic positive *Aeromonas* isolated in the present study were from the fish origin (83.33%) as compared to chicken (16.67%) which is supported by the findings of Sanyal *et al*. [54] and Sharma *et al*. [33].

## Conclusion

Hemolysin which is considered to be a major virulence factor in *Aeromonas* infection was detected in 18 (58.06%) isolates. The activity was observed on sheep blood agar plates which confirmed that hemolysis was present. *Aeromonas* are recognized to cause variety of diseases in both animals and man. In humans, they are associated with intestinal and extra-intestinal infections. These have been isolated from a wide variety of fresh foods which get contaminated by infected water, animal feces and by infected symptomatic and asymptomatic food handlers. Ability of the *Aeromonas* spp. to grow at refrigerated temperatures and occurrence of food-borne strains with enterotoxigenic capabilities to strains from gastroenteritis is a cause for concern. The available information and data on its prevalence are scanty and it is in this context that, the present study was envisaged with a view to isolate and characterizes the *Aeromonas* pathogen in fish and chicken. Comprehensive enteric disease surveillance strategies, prevention and education are essential for meeting the challenges in the years ahead. It is important for us to promote the value of enteric cultures when patients have gastrointestinal illness or bloody diarrhea or when multiple cases of enteric disease occur after a common exposure. The issue of surveillance must be among our highest priorities and understanding the role that of fish and chicken-borne disease plays as an emerging infectious disease problem.

## Authors’ Contributions

Each and every author has contributed the relevant literature in preparation of this work of review. PKP and co-authors carried out their investigations and experimntations on the mentioned topic. All authors read and approved the final manuscript.
